# Commentary: restarting NTD programme activities after the Ebola outbreak in Liberia

**DOI:** 10.1186/s40249-017-0272-8

**Published:** 2017-05-01

**Authors:** Brent C. Thomas, Karsor Kollie, Benjamin Koudou, Charles Mackenzie

**Affiliations:** 1 Centre for Neglected Tropical Diseases (CNTD), Liverpool School of Tropical Medicine, Liverpool, UK; 2 Neglected Tropical Disease, Non-Communicable Disease and Eye Health Group, Ministry of Health, Monrovia, Liberia

**Keywords:** West Africa, Ebola, Neglected tropical diseases, Mass drug administration, Health systems

## Abstract

It is widely known that the recent Ebola Virus Disease (EVD) in West Africa caused a serious disruption to the national health system, with many of ongoing disease focused programmes, such as mass drug administration (MDA) for onchocerciasis (ONC), lymphatic filariasis (LF) and schistosomiasis (SCH), being suspended or scaled-down. As these MDA programmes attempt to restart post-EVD it is important to understand the challenges that may be encountered. This commentary addresses the opinions of the major health sectors involved, as well as those of community members, regarding logistic needs and challenges faced as these important public health programmes consider restarting. There appears to be a strong desire by the communities to resume NTD programme activities, although it is clear that some important challenges remain, the most prominent being those resulting from the severe loss of trained staff.

**Electronic supplementary material:**

The online version of this article (doi:10.1186/s40249-017-0272-8) contains supplementary material, which is available to authorized users.

## Multilingual abstracts

Please see Additional file 1 for translations of the abstract into the five official working languages of the United Nations.

## Background

The Ebola Virus Disease (EVD) outbreak that devastated Liberia, Guinea and Sierra Leone from late 2013 to 2015 was assessed in early 2015 to have infected 28 637 individuals, and tragically caused 11 315 reported deaths (513 of which were health workers) [1]. In addition to the human cost, the outbreak caused serious disruption to the health and social infrastructure at all administrative levels in all these countries, as well as promoting a unfortunate mistrust of health authorities [2, 3]. This disruption to the health systems resulted in ongoing public health programmes, such as those for neglected tropical diseases (NTDs) and malaria, being suspended or drastically limited through much of this period. Before the EVD outbreak Liberia, Guinea and Sierra Leone were all successfully implementing various NTD programmes and moving steadily forward. As a number of the NTD programmes are aimed at ‘elimination as public health problems’ through interruption of transmission of infection, any interruption to annual treatments causes significant delay in the continued progress towards the goals set out by the World Health Organisation (WHO) NTD road map for these diseases [4]. Thus it is vital that mass drug distribution (MDA) restarts as soon as is possible and the activities of the newer NTD programmes, such as the chemotherapy for schistosomiasis need to be scaled up [4, 5]. In addition, patients suffering from the chronic morbidities associated with NTDs also need to be provided with basic health care.

In mid-2015, as the EVD crisis was waning in Liberia, many of the county officials reported to the national NTD director that both district authorities and community members were asking when the lymphatic filariasis MDA might restart, and indicating that they recognised the benefits MDA had provided and that they wished to regain these benefits. These questions were often posed to the NTD director and lymphatic filariasis (LF)/onchocerciasis (ONC) coordinators by the community personnel even though the medical team efforts at the time were still actively engaged in implementing the vital EVD control measures. An initial canvassing of Ministry of Health (MoH) officials, of County Health Teams, and of various key funding partners, regarding a return to active NTD programmes implementation indicated that it was likely that there would be challenges facing such a restart (Table 1).

**Table 1:** Concerns voiced by Ministry of Health and Implementing Partners regarding the NTD programmes post-EVD and proposed strategies to overcome them^a^

Concerns	Potential Consequences	Mitigation Strategies
Lack of health personnel	Reduction in health care personnel (due to migration and EVD deaths) may reduce ability of countries to quickly re-implement large scale health programmes	A focus on training of health staff (pre and post-service) and the procurement of further funding for staff.
Lack of trust in the health system	A lack of trust in the system could lead to reduced participation (compliance being essential for NTD success) and consequently the critically needed volunteer distributors may not be available. This is regarded as potentially the most debilitating challenge.	Using methods of social mobilisation that worked during the EVD outbreak to improve trust and community ownership of the programmes and activities. Success of using this approach during EVD suggests that is likely to be successful in regaining trust.
Appropriateness of the social mobilization material?	Existing materials and strategies may not be optimized to ensure the people’s understanding and trust in the programmes post-EVD	Review and edit existing material and methods used to incorporate lessons learned from EVD and thus improve social mobilization.
Disruption to the logistical management of MDA	There may be a reduced ability to efficiently transport the required drugs and materials to the various distribution centres	Focus by central administration to improve the logistics and communication infrastructure; important as this impacts all public health programmes.
Misperceptions of individuals with chronic conditions caused by NTDs	There may be reluctance for patients with chronic conditions (such as hydrocele and lymphoedema, buruli ulcer and leprosy) to become involved in morbidity management activities due to fears related to their experiences during the EVD outbreak and continued social stigma	Development of an integrated disease management plan to identify improved ways of reaching patients and providing access to treatment; this should be been focused on increasing disease awareness and understanding in the communities.

^a^A survey of Ministry of Health (MoH) officials, a majority of County Health Teams, and various key funding partners, whilst EVD control activities were underway

## A more detailed understanding

As the NTD programmes restart two key areas of information are needed, not just in relation to a single geographical area but also across the whole county: a) Acceptance? - Would a reactivated programme be accepted by the population? Would the people take the various medications and follow the needed morbidity control procedures? and b) Logistically? - Are the needed distribution and support systems in place, and also are the required support personnel available? Additionally, a better understanding of the costs associated with a restart of the programme needs to be gained, and how best any of the new facilities and structures that were previously put in place to control EVD could be used in a renewed NTD effort?

The opinions and comments of county authorities were provided to the NTD staff at a time when the EVD activities were winding down; these are individuals who had direct contact with the district staff and community members, and thus well aware of the situation in their local areas. Additionally, this information was reinforced through direct contact with community members by the national staff during their field visits for routine EVD control activities, and similarly by implementing partners working in the field. In many instances community members often actively approached MoH vehicles they recognised in public and held discussions with these health officials.

## Key findings

The feedback received from the large number of County and District authorities suggested that the overall opinion of the population was that the NTD programmes should indeed restart. This positive attitude to restarting was further confirmed by the many comments made to the national NTD staff by both community health workers and residents during the period when the EVD work was still ongoing. Comments such as “When will the programme be resuming?”, and “Have you brought the drugs for MDA?”, were not uncommon.

It is clear that any questions pertaining to the logistic ability of the country to restart the MDA programmes will be complicated. Although many of the personnel who previously been involved in MDA activities had either moved to other work areas or had unfortunately lost their lives in the outbreak, there still appears to be a willingness to restart and that people would indeed participate, as found by Bogus et al. [6]. The MoH/NTD Programme believes that in many districts there are adequate numbers of staff ready and willing to implement these programmes, although it is clear that considerable retraining of the MDA procedures for former staff, and basic training of new staff, would be needed.

## Discussion

Although our conclusion that MDA programmes in Liberia should be restarted as soon as possible, or at least rebuilt, comes from comparatively informal health system based data and from unstructured conversations with community members, it does appear that this is a true representation of the country wide opinion in Liberia. From the information that our conclusions are drawn from indicates that the NTD programmes are well recognised and valued by the community and within the national health system. Personal communications from colleagues and partners working in the two neighbouring countries also heavily affected by EVD, suggest that the situation there is similar. Guinea reported that their NTD programme is returning to pre-EVD operational levels with the LF MDA in late 2015 reaching 65% coverage in all LF endemic districts (Goep, pers. comm.).

The first NTD programmes to be restarted post-EVD are those for LF and ONC; these two programmes have been the longest running and are well recognised within communities. The NTD integrated programme has been built on the experiences of the original ONC programme which began in 2000, and thus these two NTDs require only minimal additional re-training to restart. It was noted by the Liberian NTD director that the schistosomiasis NTD programme, would in all likelihood require more detailed attention and extensive social mobilisation than that needed for LF/Oncho due to community apprehension. A recent African Regional Office (AFRO) mapping activity of school children demonstrated that there was apprehension at the community level with this programme due to confusing the clinical side effects sometimes encountered during praziquantel (PZQ) treatment with being the possible return of EVD symptoms (Table 2).

**Table 2:** Key comments and concerns raised by health staff and community members within Liberia pertaining to the restart of the NTD programme in a Post-Ebola setting^a^

	Comments by health workers and community members
Positive statements	□ County health authorizes contacting NTD team expressing desire for programme restart before EVD crisis had ended. □ Health workers expressed a desire to staff (when in the field) for the LF/Oncho programmes to resume. □ Community members asking NTD staff if they had come with the MDA drugs. □ The Liberian national NTD director express the view that the majority of people in endemic communities want activities to restart as soon as possible.
Voiced concerns	□ Concerns were expressed regarding possible reduced compliance within communities due various factors (mistrust of system, lack of distributors, etc. □ Parents expressed concern regarding the post-treatment PZQ reactions in children; these reactions were sometimes confused with being the much feared clinical signs of Ebola returning (this was seen during the AFRO SCH mapping activities in July 2015 shortly after new cases of EVD had been reported).

^a^Data collected from County and District Health Teams and communities during EVD control activities

Restarting the MDA programmes will in all likelihood be achieved by a well-planned approach that provides for the specific needs of each NTD’s programme; this focused approach must include strategies to overcome potential challenges that have been identified by the country and partners (Table 1). For example, active pre and post service training where needed, and incorporating the lessons that were learnt whilst responding to the EVD outbreak (such as improved methods of social mobilisation and community engagement). Increasing financial support is also needed to improve the reach and capacity of the programmes, the coverage. Early reports from the NTD programmes in Liberia and Guinea indicate that the mitigation approaches suggested here are being successfully implemented as MDA restarts in these countries, and thus the initial views of the MoH regarding the population’s desire for the programme appears to be correct. It appears that the impact on NTDs lost due to the EVD outbreak could now be regained sooner rather later.

## Conclusion

It is clear from our investigation that communities and the health system authorities would clearly like the NTD programmes, in particular the LF/Oncho programme, to restart as soon as possible. The benefits provided by these programmes are well recognised by these sectors as is underscored by the feedback received by the MoH staff summarised in this communication. There are, however, some important key challenges, such as lack of personnel, mistrust with the health system and misperceptions of individuals with chronic conditions, which need to be addressed to ensure the programmes can restart and scale-up successfully. To overcome these additional training, social mobilisation and support (both technical and financial) is needed.

As a result of this initial survey of the situation regarding NTD implementation, the Ministry and NTD external partners feel it is important now to obtain a wider and deeper understanding of the post- EVD situation to decide what is the best course of action at the present time for restarting these important public health initiatives; a comprehensive survey of the situation in being established.

## Additional file

Additional file 1: Multilingual abstracts in the five official working languages of the United Nations. (PDF 848 kb)

## Abbreviations

AFRO: African regional office
EVD: Ebola virus disease
FPSU: Filarial programme support unit
LF: Lymphatic filariasis
MDA: Mass drug administration
MoH: Ministry of health
NTD: Neglected tropical disease
ONC: Onchocerciasis
PZQ: Praziquantel
