# Supplementary material for: Commentary: restarting NTD programme activities after the Ebola outbreak in Liberia
Source: Infect Dis Poverty. 2017 May 1;6:52. doi: 10.1186/s40249-017-0272-8 (PMC5410695; doi:10.1186/s40249-017-0272-8)
Supplement: Additional file 1: — Multilingual abstracts in the five official working languages of the United Nations. (PDF 848 kb) [file 40249_2017_272_MOESM1_ESM.pdf]

Translation of the abstract into the five official working languages of the United Nations

التعليق: استئناف أنشطة البرنامج الأمراض الاستوائية المهملة بعد تفشي الايبولا في ليبيريا

برنت جيم توماس، كارسور كولي، بنيامين كودو، تشارلز ماكنزي

ملخص

من المعروف على نطاق واسع أن مرض فيروس الإيبولا الذي انتشر مؤخرا في غرب أفريقيا تسبب في اضطراب خطير في النظام الصحي الوطني، ومع محاولة العديد من برامج المرض السارية التي تستهدف أمراضا محددة، مثل برامج توزيع الدواء على نطاق واسع لداء كلابية الذنب، وداء الخيطيات اللمفاوية والبلهارسيا، يجري مع وقف العديد منها أو تقليصها. ومع تسعى هذه البرامج لتوزيع الدواء على نطاق واسع إلى استئناف نشاطها بعد مرض فيروس الإيبولا من المهم أن نفهم التحديات التي يمكن مواجهتها. يتناول هذا التعليق آراء القطاعات الصحية الرئيسية المعنية، وكذلك أفراد المجتمع، بخصوص الاحتياجات اللوجستية والتحديات التي تواجهها هذه البرامج الهامة للصحة العامة الساعية لاستئناف نشاطها. ويبدو أن هناك رغبة قوية من جانب المجتمعات المحلية لاستئناف أنشطة برنامج الأمراض الاستوائية المهملة، على الرغم من أنه من الواضح أنه لا تزال هناك بعض التحديات الهامة، أبرزها تلك الناجمة عن العديد الشديد في الموظفين المدربين.

Translated from English version into Arabic by Mahmoud Sami, through

TRANSLATORS
WITHOUT BORDERS

评论：利比里亚后埃博拉时代重启被忽视热带病项目

Brent C. Thomas, Karsor Kollie, Benjamin Koudou, Charles Mackenzie

摘要

众所周知，西非近来的埃博拉暴发对各国卫生系统严重破坏，诸如盘尾丝虫病、淋巴丝虫病和血吸虫病的群体性化疗等正在进行的疾病控制项目被终止或缩减。随着后埃博拉时代这些群体性化疗项目的重启，了解可能面临的挑战很重要。本评论阐述了相关的主要卫生部门以及社区成员关于重启这些重要的公共卫生项目时的物质需求和面临的挑战方面的看法。很明显社区对重启被忽视热带病的防治具有强烈的需求，尽管还有一些重要的挑战，其中最重要的是训练有素的工作人员的严重流失。

Translated from English version into Chinese by Men-Bao Qian, edited by Pin Yang

CHINESE CENTER FOR DISEASE CONTROL AND PREVENTION
NATIONAL INSTITUTE OF PARASITIC DISEASES

Commentaire : Reprise des activités du programme relatif aux maladies tropicales négligées (MTN) après l'épidémie Ebola au Libéria

Brent C. Thomas, Karsor Kollie, Benjamin Koudou, Charles Mackenzie

Résumé

Nul n’ignore que la récente maladie à virus Ebola (EVD) en Afrique de l'Ouest a entraîné une grave perturbation dans le fonctionnement du système national de santé, de nombreux programmes de santé en cours tels que l’administration massive de médicaments (AMM) contre l'onchocercose (ONC), la filariose lymphatique (FL) et la schistosomiase (SCH) ayant été soit suspendus soit réduits. À l’heure où l’on tente de faire redémarrer ces programmes d’AMM après l'épidémie, il est important de comprendre les difficultés susceptibles de se présenter. Ce commentaire examine les opinions des principaux secteurs de santé impliqués, ainsi que celles des membres de la communauté, en ce qui concerne les besoins logistiques et les défis auxquels il faudra faire face pour faire redémarrer ces programmes importants de santé publique. Il semble qu’il y ait un fort désir de la part des communautés de reprendre les activités du programme relatif aux maladies tropicales négligées (MTN) quoiqu'il soit clair que certains défis majeurs demeurent, principalement ceux résultant de la perte importante de personnel qualifié.

Translated from English version into French by Carol Molter, through

TRANSLATORS
WITHOUT BORDERS

Комментарий: возобновление мероприятий по программе «Забытые тропические болезни» после вспышки Эболы в Либерии

Брент Томас (Brent C. Thomas), Карсор Колли (Karsor Kollie), Бенджамин Куду (Benjamin Koudou), Чарльз Маккензи (Charles Mackenzie)

Аннотация

Широко известно, что недавняя вспышка болезни, вызванной вирусом Эбола (БВВЭ), в Западной Африке привела к серьёзным нарушениям в работе национальной системы здравоохранения; при этом были приостановлены или сокращены многие текущие программы, ориентированные на конкретные заболевания, такие как широкомасштабное лечение онхоцеркоза, лимфатического филяриатоза и шистосомоза. Сейчас, когда делается попытка возобновить эти программы широкомасштабного лечения после БВВЭ, важно понимать ожидающие трудности. Данный комментарий отражает мнения представителей основных затронутых секторов здравоохранения и общественности относительно материально-технических нужд и стоящих перед ними проблем, в то время как рассматривается возобновление этих важных здравоохранительных программ. Как представляется, общины выражают сильное желание возобновить мероприятия по программе «Забытые тропические болезни», несмотря на очевидное наличие существенных трудностей, самая серьёзная из которых является следствием утраты огромного числа обученных сотрудников.

Translated from English version into Russian by Natalia Potashnik, through

Comentario: Reinicio de las actividades del programa de Enfermedades Tropicales Desatendidas (NTD) tras el brote de Ébola en Liberia

Brent C. Thomas, Karsor Kollie, Benjamin Koudou, Charles Mackenzie

Resumen

Todo el mundo sabe que la reciente Enfermedad por Virus Ébola (EVD) en África Occidental ha causado una seria alteración en el sistema nacional de salud y que muchos de los programas actuales enfocados en la enfermedad están suspendidos o reducidos, como la administración masiva de fármacos (MDA) para la oncocercosis (ONC), la filariasis linfática (LF) y la esquistosomiasis (SCH). A medida que estos programas de MDA intentan reiniciar el post-EVD, es importante comprender las dificultades que se pueden encontrar. Este comentario se refiere a las opiniones de los principales sectores de salud implicados y de los de los miembros de la comunidad respecto a las necesidades logísticas y a los problemas que surgen como consecuencia del reinicio de estos importantes programas de salud pública. Parece que las comunidades quieren reanudar las actividades del programa de NTD, aunque es evidente que siguen existiendo problemas importantes, entre los que destaca la severa pérdida de personal capacitado.

Translated from English version into Spanish by Amparo Muñoz, through
